# Comparative Micro-CT Analysis of Minimally Invasive Endodontic Systems Using 3D-Printed Replicas and Natural Teeth

**DOI:** 10.3390/ma17215279

**Published:** 2024-10-30

**Authors:** Ana Ramírez-Muñoz, Marta Escribano-Capdevila, Natalia Navarrete, Gaya C. S. Vieira, Marcela Salamanca-Ramos, P. S. Ortolani-Seltenerich, José Aranguren, Alejandro R. Pérez

**Affiliations:** 1 Department of Endodontics, Rey Juan Carlos University, 28032 Madrid, Spain; anaramimu96@gmail.com (A.R.-M.); martaescricap@gmail.com (M.E.-C.); natnavarrete@hotmail.com (N.N.); marce0415@gmail.com (M.S.-R.); josearanguren@hotmail.com (J.A.); 2 Department of Conservative and Prosthetic Dentistry, Universidad Complutense, 28040 Madrid, Spain; 3 Department of Clinical Dentistry, Faculty of Biomedical and Health Sciences, Universidad Europea de Madrid, 28670 Madrid, Spain; 4 Private Practice in Porto, 4400-239 Vila Nova de Gaia, Portugal; dragayacarolina@gmail.com; 5 Department of Dental Pathology and Therapeutics, Faculty of Dentistry, Catholic University of Murcia, 30107 Murcia, Spain; psortolani@gmail.com

**Keywords:** micro-computed tomography, unprepared root canal walls, 3D-printed tooth, minimally invasive instruments, chemomechanical preparation

## Abstract

(1) Background: This study aimed to compare the shaping abilities of modern minimally invasive endodontic systems using natural teeth and 3D-printed resin replicas. These replicas offer a standardized approach for studying root canal preparation while eliminating the variability and scarcity of natural teeth. (2) Methods: Eleven mandibular molars with Vertucci class IV anatomy and their 3D-printed replicas (*n* = 132 canals) were scanned using micro-CT before and after preparation with six rotary systems. Shaping abilities were assessed by comparing volume, surface area, and unprepared areas between natural teeth and their 3D replicas, focusing on the apical third. Statistical analysis included the Shapiro–Wilk test to assess data normality and ANOVA and *t*-tests to compare different endodontic systems. (3) Results: Both qualitative and quantitative analyses revealed high similarity between natural teeth and 3D replicas. No significant differences in volume or surface area were found except in the apical third, where 3D replicas showed slightly larger increases in volume. (4) Conclusions: 3D resin replicas closely mimic natural teeth and provide a practical tool for assessing the shaping abilities of endodontic systems. This study demonstrates that 3D-printed models are suitable for endodontic research, offering a standardized and accessible alternative to natural teeth.

## 1. Introduction

The main microbiological goal of endodontic treatment is to reduce the microorganisms inside the canal in order to control apical periodontitis [1]. An essential step in achieving this goal is chemomechanical preparation, which allows for proper root canal cleaning, shaping, and disinfection [2].

However, it has been observed that our main challenge during root canal instrumentation and irrigation is the internal anatomy. Micro-computed tomography (micro-CT) studies have shown that 26–45% of root canal walls remain uninstrumented after chemomechanical procedures [3,4]. Pulp remnants, necrotic tissue, and bacteria may persist in these areas and cause endodontic treatment failure [4].

It is essential to compare different modern instrumentation systems under difficult anatomical conditions in order to develop new instrumentation strategies to remove infected dentin without increasing the risk of leaving the tooth susceptible to fracture.

Nowadays, conservative systems are available on the market. Most of them follow the current philosophy of minimally invasive endodontics (MIE), which is based on wearing down the dentin as little as possible [5] to avoid the wear of the pericervical dentin, which is located 4 mm (mm) above and 4 mm below to the bone crest [6]. Preserving the pericervical dentin is essential to achieve a better distribution of masticatory forces and thus prevent tooth fractures [7].

Another factor to consider in fracture resistance is the apical diameter and taper of the instrument used. It has been observed that instruments with a greater taper lead to higher wear of healthy dentin [8]. In addition, studies have shown that using apical diameters larger than 25/.04 significantly reduces resistance to root fractures [9,10].

However, it is important to maintain a balance between increasing the apical diameter of the preparation to optimize disinfection [11] and increasing the chances of success [12] while avoiding excessive dentin wear to prevent fracture of the endodontically treated tooth.

The study of the behavior of the rotary instruments used during chemomechanical preparation has so far been performed on extracted natural teeth. Still, due to the anatomical diversity of the root canal of each tooth [13], it is difficult to obtain a large number of samples to compare many instrumentation systems in the same study. In addition, it is challenging to collect and store human teeth; their use poses potential risks for cross-infection [14], and the standardization process is complicated.

The use of simulated canals in plastic blocks has been proposed as an alternative [15]. Nonetheless, these only simulate a single root canal instead of an entire tooth with a complex anatomy. In recent years, several factory-made models that are realistic and standardized have come onto the market [16]. Nevertheless, they are costly, the choice of tooth types is limited, and the differences between the manufacturing processes and the materials used by each brand are very large [16].

The use of replicas of natural tooth anatomies may overcome these limitations. Three-dimensional printing (3D) is a rapidly developing technology that has gained wide acceptance in dentistry [17]. As more materials become available, 3D-printed teeth offer unprecedented opportunities to create new, individualized models that can be used in many fields of endodontic research, such as irrigation, obturation [18], and chemomechanical preparation [19]. Studies using 3D resin replicas [16,20] concluded that the accuracy of the printing process is suitable for the production of tooth replicas for endodontic education and easier standardization.

An important drawback of printed teeth is that their properties differ from those of dentin [21]. Their hardness and radiopacity are not comparable to those of natural human teeth [21]. However, they are suitable for mimicking all aspects of endodontic treatment [22] and have the potential to compare different instrumentation systems.

One recent study compared two instrumentation systems using 3D resin teeth, but only one natural tooth was used to print the 3D replicas [19]. To our knowledge, no studies compare, with micro-CT, the root canal anatomy of the 3D replica teeth with that of natural teeth quantitatively and qualitatively.

To address these limitations, this study compared the shaping abilities of six conservative instrumentation systems on natural teeth and their 3D-printed resin replicas. The null hypothesis was that there would be no significant differences in shaping abilities, measured by volume and surface area, between the natural teeth and 3D-printed replicas across the full canal length and the apical third. The characteristics regarding each system’s apical size, taper, alloy, and cross-section can be found in the Supplementary Table S1.

## 2. Materials and Methods

### 2.1. Sample Selection

The institutional ethics committee approved the study (protocol 1702202308023). The sample size was calculated with the software G*Power 3.1 (Heinrich Heine Universität, Duesseldorf, Germany) with an alpha error of 0.05 and a power of 95%. It was found that at least 12 root canals were required in each group. From an initial group of 60 natural teeth, 11 mandibular molars extracted for reasons unrelated to this study were selected, resulting in 22 root canals. The following inclusion criteria were applied: First mandibular molars with mesial canals with Vertucci’s [13] classification type IV and moderately curved (<20°). Teeth with root caries, internal resorptions, calcifications, vertical fractures, or immature root apices were excluded.

The initial selection was based on periapical radiographs in the buccolingual and mesiodistal directions, and then the anatomies were confirmed by micro-CT scans.

### 2.2. Micro-CT Scanning

Natural tooth and 3D replica samples were scanned before and after chemomechanical preparation on the Phoenix Vitomex S240 micro-CT (General Electric, Boston, MA, USA).

The teeth specimens were digitized with the following parameters: 20.0 mm isotropic resolution, 155 kV, 190 mA, 360° rotation around the vertical axis, and a 0.2 mm thick filter, resulting in a scan time of approximately 20 min per tooth. 3D replicas, in turn, were scanned using 125 kV and 90 mA because, with higher parameters, it was not possible to visualize its external and internal structures. The other parameters were the same as described above. The teeth were reconstructed using the Phoenix-x 3D program (General Electric, Boston, MA, USA). A total of 1450 images per tooth was created with the following parameters: ring artifact correction of 7, beam hardening correction of 60%, and smoothing of 7.

A stereolithography (STL) file was created from the original reconstruction for internal visualization of each anatomy using 3D Slicer 5.0.3 software http://www.slicer.org (accessed on 30 March 2023). These files were then used to print the 3D replicas (see below).

### 2.3. Manufacturing of 3D Replicas in Resin

The natural teeth were first prepared by reducing the crown up to 3 mm to the cementoenamel junction. After the initial micro-CT scan, the mesial roots of each natural tooth (*n* = 11) were printed. Initially, the STL files of the teeth were inserted into the AnyCubic PhotonWorkshop software https://www.anycubic.es (accessed on 1 April 2023) to determine the appropriate position and necessary supports for the subsequent printing. Then, eleven replicas of the same anatomy were positioned in the vat of the Anycubic Photon Mono M5s printer (Anycubic Technology Co., Shenzhen, China), which can manufacture models with a resolution of up to 10 μm. The file created was then saved on a USB stick (SanDisk, Milpitas, CA, USA) and inserted into the machine for printing. The printer’s vat was filled with 200 milliliters (mL) of white UV-Tough resin (Anycubic Technology Co., Shenzhen, China) with a 365–405 nanometers wavelength to print the replicas. The printing time was approximately 1 h and 30 min. After manufacturing, the replicas were washed in 70% alcohol using the Anycubic Wash & Cure Plus device (Anycubic Technology Co., Shenzhen, China) to remove excess resin fillings in the canals and subjected to a polymerization process for 20 min using the same machine for the final curing. Four replicas were used so that the operator could practice prior instrumentation. Eleven 3D resin replicas were included in each group (n = 66) to compare the different systems. A total of 22 root canals’ natural teeth and 132 3D mesial canal resins were included in the study.

### 2.4. Chemomechanical Preparation of Natural and 3D Teeth

The teeth and 3D replicas were mounted vertically in resin blocks (Zhermack, Badia Polesine, Italy) to create a vapor-lock effect [23]. A hand K-type file size 10 (Dentsply Tulsa Dental, Charlotte, NC, USA) was inserted into the canal until it was visible through the apical foramen. Then, 1 mm was subtracted from this length to determine the working length (WL). Instrumentation was performed by a single operator using a surgical microscope (Carl Zeiss, Berlin, Germany) during all procedures. Each rotary instrument was used to prepare a maximum of four root canals, following the manufacturer’s recommendations. The file systems were driven by an endodontic motor (Eighteeth, Changzhou, China) and performed in and out movements with an amplitude of 3 to 4 mm until WL was achieved without brushing. After use, the instruments were cleaned with sterile gauze and alcohol.

An initial canal irrigation protocol was performed in all instrument groups with 2 mL of 2.5% sodium hypochlorite (NaOCl) with a Navitip 30 G needle (Ultradent, South Jordan, UT, USA) placed 3 mm from the WL. Rinsing with 2 mL NaOCl was performed between instruments, and patency was established with a hand K-type file size 10. At the end of instrumentation with each system, a final irrigation protocol was performed until 10 mL NaOCl was reached in all groups.

The Race-Evo (RE) instrument system (FKG Dentaire, La Chaux-de-Fonds, Switzerland) was used to prepare natural and 3D resin teeth. It was used in continuous rotation at 1000 revolutions per minute (rpm) and a torque of 1.5 Newtons per centimeter (N/cm). Once the WL was reached, the file was removed from the canal, and the same protocol was used for the following sequence of files in the system: RE1 (15/.04), RE2 (25/.04), and RE3 (30/.04).

The Rotate system (RO) (VDW, Munich, Germany) was used at 350 rpm and a torque of 1.3 N/cm. Once the WL was reached, the protocol followed the file sequence recommended by the manufacturer: 15/.04, 25/.04, and 30/.04.

The Slim Shaper instrument files (SS) (Zarc4endo, Gijón, Asturias, Spain) were used with a continuous rotation speed of 500 rpm and a torque of 3 N/cm. Once the WL was reached, the protocol was continued according to the file sequence recommended by the manufacturer: ZS1 (15/.02–06), ZS2 (20/.04), and ZS3 (25/.04). Finally, the apical third was enlarged with the Z30 (30/.03) instrument from Apical Shaper (AS) (Zarc4endo, Gijón, Asturias, Spain) at 500 rpm and a torque of 3 N/cm.

The TruNatomy (TA) instrumentation system (Dentsply Sirona, Ballaigues, Switzerland) was used with a continuous rotation speed of 500 rpm and a torque of 1.5 N/cm. Once the WL was reached, the protocol was continued according to the manufacturer’s recommended file sequence: Orifice Modifier (20/.08), Glider (17/.02), Prime (26/.04), and Medium (36/.03).

Due to the differences between the Prime and Medium files in the TA system, another group with AS was added to observe if there was a difference between the use of 30/.03 and 36/.03 files. The instrumentation started with the Orifice Modifier instrument, followed by the Glider instrument (17/.02), the Prime file (26/.04), and finally, the apical segment of the canal was increased with the Z30 file (30/.03) of the AS system.

The last group for root canal preparation was the Blueshaper-Pro (BP) (Zarc4endo, Gijón, Asturias, Spain) plus AS. Instrumentation was performed according to the manufacturer’s instructions: Z1 (14/.02–10), Z2 (17.02–10), Z3 (19.06), and Z4 (25.06) at 500 rpm and 4 N/cm AS Z30 (30.03) was used to complete the apical enlargement.

At this point, all samples were scanned again with the micro-CT.

### 2.5. Micro-CT Analysis

#### 2.5.1. Qualitative Assessment

The natural teeth and the 3D replicas were compared by showing 20 micro-CT images of the canal anatomy of the natural teeth and their 3D replica before instrumentation to two experienced endodontists in a separate darkroom. Both reviewers independently evaluated the same set of images for all samples to ensure a consistent assessment. This approach was chosen to reduce individual bias and provide a reliable qualitative comparison between the natural teeth and their 3D replicas.

The images were randomly inserted in pairs into the PowerPoint program 16.16.27 (Microsoft, Redmond, Washington, DC, USA) without identifying the natural tooth and the 3D replica. First, the reviewers had to indicate which images corresponded to the natural teeth and which to the 3D teeth. Then, they were asked to give a score from 1 to 3 according to the anatomy of each tooth, as follows: 1. Was “not similar” if the root canal was completely different in size and shape from the 3D canal and could be easily distinguished from the other; 2. Was “similar” if the natural main root canal was similar to the 3D canal but lacked some anatomical complexities (lateral canals, isthmuses, or ramifications) in the 3D canal and was easy to distinguish; or 3. “Very similar” if the main root canal and the 3D root canal were precisely the same in shape and size, both had anatomical complexities and were difficult to differentiate. Finally, the data were tabulated in an Excel spreadsheet (Microsoft, Redmond, Washington, DC, USA) for subsequent statistical analysis.

#### 2.5.2. Quantitative Validation and Shaping Ability Analysis

The validation of the shaping in 3D replicas was performed by comparing the initial and final volume of the root canals as well as the surface, the unprepared areas, and the remaining dentin/resin between the natural teeth and 3D replicas after chemomechanical preparation with RE system. After the reconstruction of the images obtained from the initial and final micro-CT scans of the natural teeth and the 3D resin teeth, Slicer 5.0.3 software was used for the coregistration of the 3D models with a custom combination of a rigid registration module based on image intensity similarities with accuracy larger than one voxel. Then, the volume (mm^3^) and surface area (mm^2^) before and after preparation of the full canal (10 mm) and the apical third segment (4 mm) were calculated using ImageJ 1.50d software (National Institutes of Health, Bethesda, MD, USA). The same program was used to calculate the number of static voxels in the unprepared areas. Values were calculated by subtracting the scores of the prepared canals from those recorded by their unprepared counterparts and then transformed into percentages.

The comparative analysis between conservative systems with micro-CT in 3D resin replicas was performed by evaluating the same parameters described above after chemomechanical preparation.

The three-dimensional models were reconstructed using the open-source modeling software 3D Meshmixer 3.5.474 (Autodesk, San Rafael, CA, USA). The color green was used for uninstrumented canals, red for SS, light blue for TA, dark yellow for TA + AS, gray for RO, purple for RE 3D replica, yellow for RE natural tooth, and blue for BP.

The remaining dentin/resin was evaluated after chemomechanical preparation. 10 mm from the cementoenamel junction to the apical third was assessed using ImageJ 1.50d software. For this evaluation, the dentin and/or resin and the canal volume (mm^3^) were recorded at the beginning and after chemomechanical procedures. Since the volume of the canal increases after the instrumentation phase and the volume of the dentin/resin decreases at the same time, the dentin/resin reduction was calculated after using each instrumentation system and expressed as a percentage.

### 2.6. Statistical Analysis

The Shapiro–Wilk test was used to assess the normality of the data obtained. As the results showed normality, the ANOVA test was used to compare the volume, surface area, unprepared root canal areas, and remaining resin between the different systems (before and after preparation) on the full canal length (10 mm) and apical canal segment (4 mm). The *t*-test was used to compare volume, surface area, unprepared areas, and remaining dentin/resin between natural and 3D resin teeth. The same test examined the volume and surface area increase within groups. The chi-square test was used to analyze the reviewers’ results to compare natural and 3D teeth. The statistical program SPSS (Statistical Package for the Social Sciences 21.0; IBM Brasil, São Paulo, Brazil) with a significance level of 5% was used for all analyses.

## 3. Results

### 3.1. Qualitative Comparison of Natural Teeth and 3D Replicas

**Figure 1:**
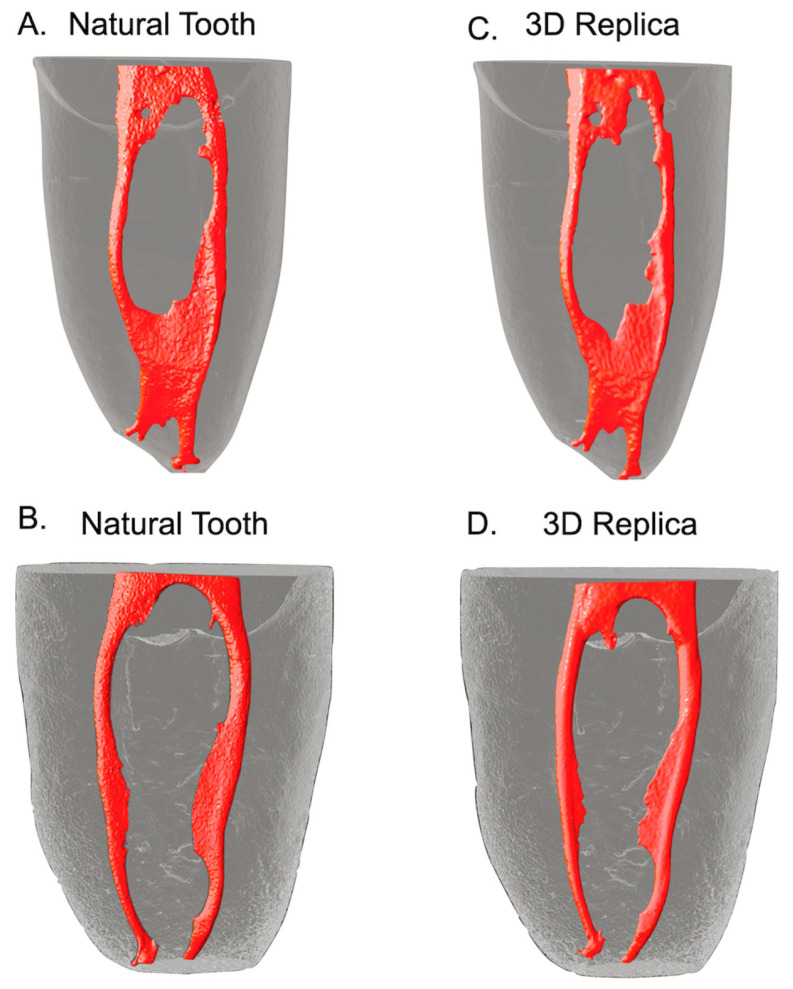
Representative 3D reconstruction models of mandibular molars for comparison. (**A**,**B**) Root canal anatomy of the natural teeth taken with micro-CT (red) and (**C**,**D**) their 3D replicas.

The evaluators did not find any natural teeth, and the 3D resin replicas were “not similar”. Observer One stated that 20% of the natural and 3D teeth were “similar”, and 80% were “very similar”. Observer two, in turn, indicated that 100% of the teeth were “similar” (Table 1) (Figure 1). Significant differences were found between the evaluators (*p* < 0.05).

Regarding the correct differentiation of the natural tooth from the 3D resin replica, of the 20 images evaluated, the reviewers could only successfully identify the samples in 20% (4/20) of the cases. Evaluator one distinguished the natural tooth from the resin replica in (4/10) 40% of the cases, and Evaluator two in (0/10) 0%. The results revealed significant differences (*p* < 0.05) (Table 1).

### 3.2. Instrumentation Comparison Between Natural Teeth and 3D Replicas

No procedural errors, such as perforations or ledges, occurred in the natural teeth or the 3D resin replicas during the chemomechanical preparation. Furthermore, no instrument fractures were observed during the procedures.

The results showed that there were no significant differences (*p* > 0.05) between the initial and final volume of the natural canals and the 3D resin replicas in the full canal length after instrumentation with the RE system (Table 2). When analyzing the apical canal segment, significant differences were observed (*p* < 0.05) in the percentage of volume increase of the natural tooth and the 3D replicas. In addition, no significant differences (*p* > 0.05) in the initial and final surface area were found between the natural teeth and the 3D resin replicas in the full canal length and the apical third segment (Table 2).

**Figure 2:**
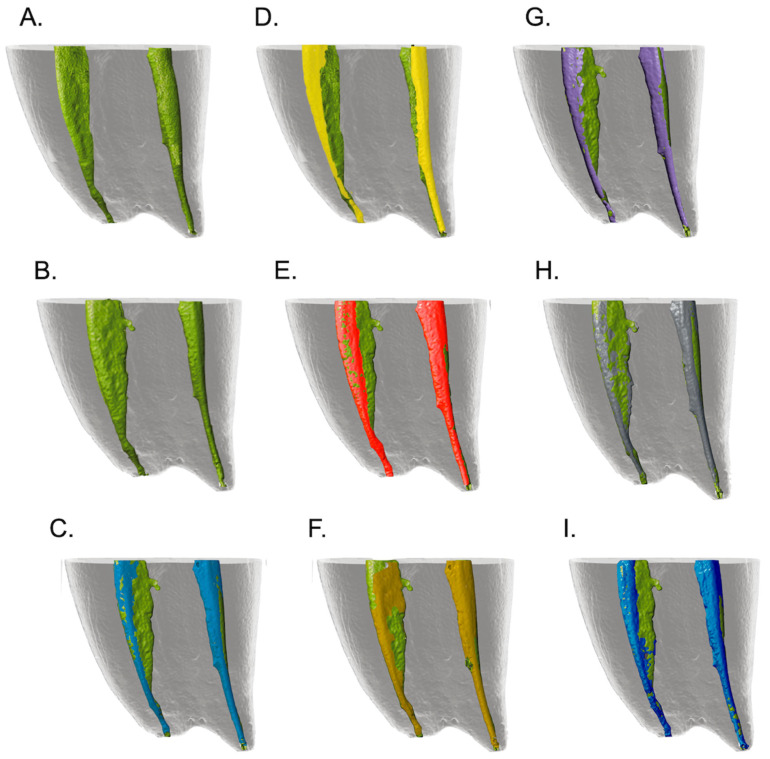
Representative images of micro-CT scans before and after chemomechanical procedures. (**A**) Preoperative image of the natural tooth (green). (**B**) Before instrumentation of 3D replicas (green). After root canal preparation of (**D**) natural tooth with RE (yellow) and 3D replicas (**C**,**E**–**I**). RE (purple), SS (red), RO (grey), TA (light blue), TA + AS (dark yellow), and BP (blue).

Regarding the unprepared root canal walls, no significant differences were found between the natural teeth and the 3D resin teeth (*p* > 0.05) in the full canal length after instrumentation with the RE system, with a percentage between 26.1% in the natural teeth and 31.5% in the 3D replicas (Figure 2). When evaluating the apical 4 mm, the unprepared areas of the natural tooth and the 3D tooth were 24.3% and 32.5%, respectively, without any differences (*p* > 0.05) between the groups.

### 3.3. Comparison Between Conservative Systems in 3D Replicas

Table 2 presents the mean, median, and range values for volume, surface area, and unprepared root canal walls across the full canal length (10 mm) and the apical third (4 mm) after preparation with each conservative instrumentation system.

No significant differences (*p* > 0.05) were observed between groups in the initial and final root canal volume and surface area for both the full canal length and apical third, except for the Blueshaper-Pro (BP) system, which showed a significantly higher percentage increase in volume over the full canal length compared to other systems (*p* < 0.05). Intragroup comparisons revealed a significant increase in volume and surface area after chemomechanical preparation in all groups (*p* < 0.05).

The percentage of unprepared areas in the total canal length was similar for all conservative rotary systems, with no significant differences (*p* > 0.05). The values were 32% for SS + AS, 30.4% for RO, 32.1% for TA, 29.2% for TA + AS, 31.5% for RE, and 28.1% for BP + AS, respectively (Figure 2).

When the apical third of the canal was analyzed, the percentage of unprepared root canal walls was 33.8% for SS + AS, 30.4% for RO, 33.4% for TA, 32.8% for TA + AS, 32.5% for RE, and 30.5% for BP + AS. There were no significant differences between the systems (*p* > 0.05).

### 3.4. Dentin/Resin Reduction

No significant differences (*p* > 0.05) were found between the instrumentation groups regarding resin reduction. The values were 12.1%, 11.9%, 11.1%, 11.4%, 11.0%, and 16.3% for SS + AS, TA, TA + AS, RE, RO, and BP + AS, respectively. The mean value of dentin reduction in natural teeth was 8.7% (Table 3).

## 4. Discussion

This ex vivo study compared the anatomy of the mesial canals of natural mandibular first molars and their replicas manufactured with a high-resolution 3D resin printer, as well as the volume, surface area, unprepared areas, and dentin/resin reduction of the root canals in natural teeth and 3D replicas in resin.

Teeth with the Vertucci classification type IV were used as they represent the most common configuration of the root canal system [24] and have independent canals, which allows a separate analysis of the effectiveness of instrumentation and increases the sample size to be studied. In addition, these canals are narrow and have little dentin thickness in the area close to the furcation [25], which makes their analysis interesting for evaluating dentin reduction and unprepared areas.

In the present study, the natural teeth and the 3D replicas were found to be very similar after the qualitative analysis, as in most cases, the evaluators could not differentiate which images corresponded to the natural tooth and which to the 3D replicas (100% for evaluator two and 60% for evaluator one). They classified all images as similar or very similar. To our knowledge, this is the first study to compare the anatomy of the root canals of 3D replicas and the corresponding natural teeth by evaluating images obtained with micro-CT.

One study [26] compared the use of natural teeth and 3D replicas regarding clinical perception in undergraduate students. In another recent study [16], scanning to produce the replicas was performed using cone beam computed tomography. This method has lower resolution compared to micro-CT technology, the gold standard for assessing the internal anatomy of teeth. So, from an anatomical point of view, the 3D replicas are comparable to natural teeth.

In this study, there were no significant differences between the initial and final volume and surface area of the natural teeth and the 3D resin replica when analyzing the full canal length, indicating a high similarity between the samples. The results are in agreement with a study [19] that showed similar results when comparing the shaping abilities of two instrumentation systems in natural teeth and 3D replicas using micro-CT.

Nevertheless, significant differences were found in the final volume percentage in the apical third between the natural tooth and the 3D replica. These findings suggest that while 3D replicas are highly accurate, the apical third’s intricate and confined anatomy may be more challenging to replicate precisely, leading to minor deviations in this area. Therefore, although 3D-printed models are reliable for most of the canal length, this limitation in the apical third should be considered when assessing their use in shaping ability studies. Future studies evaluating the apical segment in different groups of teeth should be conducted to determine whether there are differences between the 3D replica and the natural tooth in this area.

In the present study, the initial volume values of the 3D resin replicas range between 10.6 mm^3^ and 10.9 mm^3^. The values for the surface area were 103.9 mm^2^ and 104.9 mm^2^, respectively. From a standardization point of view, a remarkable similarity was found between the different 3D replicas regarding initial volume and surface area, showing the likelihood that the printer produces replicas with virtually identical anatomy.

Furthermore, no significant differences were found in volume increase after endodontic procedures, demonstrating the similarity of the MIE systems in terms of their shaping abilities. An exception was the BP system, which showed a significant increase in volume percentage compared to the other systems when the full canal length was evaluated. A larger taper (6%) could explain these differences. In addition, the use of a softer material than that of the natural tooth may have contributed to an overestimation of the volume increase after instrumentation.

Nowadays, minimally invasive endodontics has gradually gained acceptance in endodontics to maintain conservative endodontic instrumentation while respecting the anatomy of the root canal [27]. For this reason, the rotary MIE instrument systems were selected as they have similar tapers and apical diameters.

A common factor evaluated when comparing different systems is the non-instrumented areas of the root canal. They are a crucial factor during endodontic treatment as these areas can harbor necrotic tissue and bacterial biofilms that can jeopardize the final success of the treatment [2,4]. The present shaping findings revealed that the percentage of unprepared root canal walls ranged from 28.1 to 32.1% over the full canal length (10 mm) in 3D replicas and 26.1% in natural teeth. The 4 mm apical segment values ranged between 24.3% to 33.8%. These results are consistent with other studies on mandibular molars’ natural teeth, where approximately 30% or more of the root canal walls remained uninstrumented [28,29]. No significant differences were observed between instruments, probably because they have very similar tapers and apical diameters.

Curiously, there are no differences between the TA and TA-plus AS groups. An additional group was included due to the difference in apical size between the Prime File 26/.03 and Medium 36/.03 instruments. However, no instrumentation difficulties were observed when using the entire sequence of the TA system. Based on these results, it is possible to use the system as suggested by the manufacturer or to include an intermediate 30/.03 instrument during chemomechanical preparation.

One of the main shortcomings of using 3D resin replicas is their hardness, as they are completely different from dentin [21]. Although this study aimed not to compare the hardness of the 3D resin replicas with that of dentin, it is evident that the 3D resin replicas are much softer than the natural tooth, and the feeling during instrumentation was different from the natural tooth. Moreover, this was not reflected in any difficulties during the instrumentation process. No procedural errors, such as perforations or ledges, occurred during chemomechanical preparation. Nonetheless, as the instruments used have a smaller taper and are heat-treated, it is impossible to determine whether these results also apply to other systems.

It has been described that using instruments with a 4% or 6% taper is safe to reduce the risk of root fractures after chemomechanical preparation [8]. Dentin/resin reduction ranged from 8.7% in natural teeth to 11–16.3% in resin teeth, and no differences were found between groups. Most systems have similar tapers (3% or 4%), which could explain the results. One exception was the BP system, which has a 6% taper. However, the regressive variable taper could help explain the lack of difference with the other systems.

A limitation of using 3D resin replicas during root canal instrumentation is the greater reduction of resin, as it is softer than the natural tooth. Nevertheless, this was not observed in the present study because no significant differences in remaining dentin/resin were observed between the natural tooth and the 3D replica after instrumentation with the RE system. Future studies should investigate whether the use of systems using different heat treatments and tapers results in a greater reduction of resin than the natural tooth.

Regarding irrigation during chemomechanical preparation, distilled water is typically used to instrument 3D resin replicas [19], as no organic tissue can be removed. However, as this study compared a group of natural teeth with 3D resin replicas, NaOCl was chosen as the irrigant in all cases to avoid confounding variables that could influence the results.

The use of 3D resin replicas opens up many possibilities in endodontic research. Firstly, it eliminates the difficulties of matching specimens. It is possible to compare a larger number of groups with identical anatomy, and the cost per impression is low, so a large number of replicas can be printed. Furthermore, there is no risk of cross-contamination [14], and these analyses can likely be extended to other areas, such as irrigation or even root canal obturation in complex anatomies with lateral canals, isthmuses, or apical ramifications. Nevertheless, the natural tooth must remain the gold standard for evaluating shaping abilities with micro-CT. The 3D resin replicas can serve as an additional method to increase the number of groups to be compared in the same anatomies.

The results of this study support the partial rejection of the null hypothesis. While no significant differences were found in the shaping abilities of natural teeth and 3D-printed replicas over the full canal length, significant differences were observed in the apical third. This outcome suggests that, although 3D-printed replicas are reliable for most of the canal length, minor deviations in the apical third may affect the accuracy of shaping results in this region. These findings highlight the potential of 3D-printed models for endodontic research while acknowledging limitations specific to the apical anatomy.

This study has some limitations. First, 3D resin replicas have a disparity in mechanical properties compared to natural dentin. The UV-Tough resin used in this study has a Young’s modulus and ultimate tensile strength lower than that of human dentin, which typically has a Young’s modulus of approximately 18 GPa. This resin, with a wavelength range of 365–405 nm, likely has a modulus of elasticity closer to 2–3 GPa and a tensile strength of 50–70 MPa (source: Anycubic), making it considerably softer and more flexible than dentin. This difference can affect shaping results, especially in the apical third, where precise replication is challenging. Although the 3D replicas are reliable over most of the canal length, future studies could investigate whether alternative resins with properties closer to the mechanical profile of dentin can improve the accuracy in shaping studies, especially in anatomically complex areas.

Additionally, a single conservative system on natural teeth was used to validate the shaping abilities of 3D resin replicas. Future studies should include systems with different tapers and heat treatments to observe the behavior of the 3D resin replicas in different situations. In addition, during the initial manufacturing of the models, it was found that some anatomies could not be printed, mainly due to artifacts in the root canal, such as calcifications or very narrow canals. Therefore, obtaining some additional teeth to solve this problem is necessary.

## 5. Conclusions

The present study shows that the resin tooth replicas have a root canal anatomy similar to natural teeth. The method of 3D printing of resin teeth was validated to evaluate chemomechanical preparation with micro-CT using conservative instruments. In addition, the MIE systems were found to have similar shaping abilities and dentin/resin reduction behavior.

## Figures and Tables

**Table 1:** Comparison of characteristics of natural teeth and 3D-printed teeth.

Data		Observer 1	Observer 2	Total
**Anatomy**				
	Similar	20.0%	100.0%	60.0%
	Very Similar	80.0%	0.0%	40.0%
**Evaluation**				
	Correct	40.0%	0.0%	20.0%
	Incorrect	60.0%	100.0%	80.0%

**Table 2:** Micro-computed tomografic analyses before and after root canal preparation in natural and 3D-printed teeth. Data for total root canal length (10 mm) and the apical third (4 mm) expressed as a mean (median; range).

Data	Race-Evo Natural	Race-Evo 3D	Slim Shaper + AS	Trunatomy	Rotate	Trunatomy + AS	Blueshaper Pro + AS
**Total length (10 mm)**							
**Volume (mm^3^)**							
Initial	10.1 (4.9; 3.7–18.8)	10.9 (5.0; 4.3–19.8)	10.7 (5.2; 4.6–19.5)	10.8 (5.0; 4.7–19.1)	10.6 (5.4; 4.7–19.3)	10.8 (4.7; 4.3–19.2)	10.7 (4.9; 4.6–19.1)
Final	13.5 (5.7; 4.6–24.8)	13.1 (5.4; 5.9–23.8)	13.3 (5.3; 6.4–23.6)	12.9 (5.6; 5.7–24.6)	12.7 (5.4; 5.3–22.5)	12.6 (5.7; 5.6–24.5)	15.1 (6.4; 7.3–28.9)
% after preparation	33.7 (23.4; 14.7–105.1)	23.4 (13.4; 4.9–49.2)	26.2 (17.5; 8.1–60.6)	20.0 (12.1; 8.7–40.2)	18.2 (12.8; 5.6–44.1)	17.1 (9.5; 4.1–34.1)	43.4 (30.5; 15.9–110.3)
**Surface area (mm^2^)**							
Initial	96.7 (36.6; 43.2–130.2)	103.9 (38.7; 46.6–190.6)	104.0 (39.0; 46.7–191.6)	104.2 (39.1; 47.7–192.6)	104.0 (38.9; 46.7–191.6)	104.3 (38.9; 48.7–192.6)	104.9 (40.9; 47.7–200.6)
Final	117.4 (41.4; 58.6–168.7)	115.2 (39.9; 62.0–204.4)	116.1 (39.8; 66.2–200.8)	114.6 (40.0; 60.4–204.0)	114.8 (39.8; 62.4–199.8)	114.1 (41.1; 61.4–204.3)	121.9 (43.0; 69.1–219.0)
% after preparation	21.4 (16.4; 6.7–38.5)	12.2 (8.3; 5.7–32.9)	13.3 (11.1; 2.2–41.9)	11.2 (7.1; 3.5–26.7)	11.6 (9.0; 1.1–33.7)	10.1 (7.1; 3.1–26.1)	17.9 (11.1; 6.8–45.0)
**Unprepared area (%)**							
After preparation	26.1 (8.7; 10.4–43.8)	31.5 (9.6; 18.9–48.4)	32.0 (10.9; 18.6–49.2)	32.1 (13.0; 13.7–56.1)	30.4 (9.8; 11.4–46.1)	29.2 (9.8; 13.7–45.6)	28.1 (10.7; 15.4–46.2)
**Apical portion (4 mm)**							
**Volume (mm^3^)**							
Initial	2.3 (1.3; 1.6–6.9)	3.5 (1.4; 1.6–7.3)	3.3 (1.5; 1.9–7.1)	3.2 (1.2; 1.6–7.8)	3.5 (1.5; 1.5–7.2)	3.4 (1.9; 1.7–7.4)	3.6 (1.6; 1.8–7.4)
Final	3.3 (1.5; 1.0–6.7)	4.3 (1.6; 2.4–7.8)	4.4 (1.7; 2.3–8.2)	4.2 (1.6; 2.4–7.8)	4.1 (1.6; 2.2–7.7)	4.2 (1.7; 2.1–7.6)	4.6 (1.8; 2.4–8.0)
% after preparation	43.8 (25.6; 7.2–78.5)	22.3 (13.5; 3.0–51.9)	25.1 (16.3; 7.9–65.7)	21.0 (11.1; 6.1–38.6)	16.6 (11.5; 7.6–48.5)	17.6 (11.5; 6.4–44.1)	27.8 (14.7; 13.6–55.9)
**Surface area (mm^2^)**							
Initial	32.2 (9.6; 16.0–45.6)	37.3 (10.2; 21.2–56.8)	37.1 (10.4; 21.0–57.8)	37.6 (10.3; 20.7–57.1)	37.5 (10.8; 21.4–56.9)	37.4 (11.1; 21.3–57.5)	37.3 (10.2; 21.1–57.4)
Final	43.3 (12.8; 23.4–63.5)	42.8 (12.8; 22.9–62.4)	43.5 (13.5; 22.4–64.8)	43.0 (12.3; 25.7–62.2)	42.4 (12.7; 22.6–62.4)	42.4 (12.3; 21.5–61.0)	45.2 (13.4; 23.1–64.4)
% after preparation	33.8 (23.5; 9.5–48.7)	14.8 (9.9; 3.1–34.2)	16.2 (10.3; 2.2–33.9)	15.6 (8.1; 5.7–30.5)	13.5 (7.9; 5.1–26.1)	13.6 (9.7; 2.3–29.7)	21.2 (10.2; 9.8–39.2)
**Unprepared area (%)**							
After preparation	24.3 (6.4; 12.4–54.7)	32.5 (8.5; 20.9–45.2)	33.8 (11.1; 18.7–50.4)	33.4 (10.0; 17.5–51.2)	30.4 (6.9; 19.7–42.4)	32.8 (7.8; 22.8–48.4)	30.5 (8.3; 17.9–43.4)

**Table 3:** Data concerning dentin/resin reduction in natural and 3D replicas.

Data	Race-Evo Natural	Race-Evo 3D	Slim Shaper + AS	Trunatomy	Rotate	Trunatomy + AS	Blueshaper Pro + AS
**Dentin/resin reduction (mm^3^)**							
Initial	229.4 (51.4: 143.7–334.5)	234.3 (52.9; 168.7–336.7)	234.5 (53.8; 165.6–346.8)	234.2 (53.2; 165.4–345.7)	234.8 (52.6; 162.7–343.3)	234.4 (53.0; 164.8–344.7)	234.2 (53.3; 164.9–340.7)
Final	209.5 (45.8; 102.5–265.7)	207.5 (47.6; 122.9–282.0)	206.3 (48.3; 120.2–282.0)	206.6 (48.7; 121.0–286.4)	208.9 (49.8; 122.5–291.0)	208.5 (49.0; 120.8–286.4)	195.3 (42.9; 121.1–264.6)
% after preparation	8.7 (8.3; 0.9–17.5)	11.4 (8.8; 1.3–26.2)	12.1 (8.9; 1.6–27.4)	11.9 (9.3; 0.6–27.0)	11.0 (9.0; 0.0–26.0)	11.1 (8.9; 0.5–27.0)	16.3 (9.1; 4.9–34.9)

## Data Availability

The data supporting this study’s findings are available from the corresponding author upon reasonable request.

## Supplementary Materials

The following supporting information can be downloaded at: https://www.mdpi.com/article/10.3390/ma17215279/s1, Supplementary Table S1: Minimally invasive instruments characteristics.
