# Supplementary material for: Comparative Micro-CT Analysis of Minimally Invasive Endodontic Systems Using 3D-Printed Replicas and Natural Teeth
Source: Materials (Basel). 2024 Oct 30;17(21):5279. doi: 10.3390/ma17215279 (PMC11547619; doi:10.3390/ma17215279)
Supplement: Supplementary file 1 [file materials-17-05279-s001.zip › materials-3285142-supplementary.pdf]

Supplementary Table S1. Minimally invasive instruments characteristics.

System	Apical Size	Taper (%)	Speed / Torque (Rpm/Ncm)	Maximal Flute Diameter	Alloy	Cross-Section
**Rotate**	15-30	4%	300-400/1.3-2.3	1.03	Blue	S-Shape
**Race Evo**	15-30	4%	800-1000/1.5	0.89-1.11	Blue/ Electropolishing	Triangular
**Slim Shaper**	15-25	4-3%	500/3	0.80-0.84	Gold, Pink, Blue	Triangular
**Apical Shaper**	30	3%	350/2.5	0.78	Blue	Parallelogram
**Trunatomy**	17-36	3%	500/1.5	0.70-0.80	Superflex	Convex Triangular, Parallelogram
**BlueShaper Pro**	14-25	5-6%	500/4	0.88-0.91	Dual Wire	Convex Triangular
